# Supplementary material for: Impact of resource scarcity on general categorization tendency: The moderating role of perceived mutability
Source: PLoS One. 2023 Aug 23;18(8):e0286619. doi: 10.1371/journal.pone.0286619 (PMC10446183; doi:10.1371/journal.pone.0286619)
Supplement: Attachment — Submitted filename: renamed_81bb8.docx [file pone.0286619.s003.docx]

Dear Editor and reviewers:

Re: “Impact of Resource Scarcity on General Categorization Tendency: The Moderating Role of Perceived Mutability”

Great thanks for your professional review of our paper. We have benefited greatly from reading your review comments. After careful consideration and study of your comments, we have made extensive corrections to the previous manuscript.

Each comment from all reviewers has been carefully considered and responded to. To make it easier for you to read, we have organized our feedback in the following format. The reviewers' comments are listed in *italicized font*, and specific concerns have been specifically numbered. Our replies are given in normal font, in addition, corrections and additions to the manuscript are given in red font.

Below we present our responses to the specific issues raised by each reviewer.

Yours truly,

The authors

**Response to the journal requirements:**

*1. Please ensure that your manuscript meets PLOS ONE's style requirements, including those for file naming. The PLOS ONE style templates can be found at*

*https://journals.plos.org/plosone/s/file?id=wjVg/PLOSOne_formatting_sample_main_body.pdf and*

[*https://journals.plos.org/plosone/s/file?id=ba62/PLOSOne_formatting_sample_title_authors_affiliations.pdf*](https://journals.plos.org/plosone/s/file?id=ba62/PLOSOne_formatting_sample_title_authors_affiliations.pdf)

**Our response:**

Thanks for your reminder, we have changed and rechecked the format to ensure our manuscript meets PLOS’s ONE’s style requirements.

*2. Please provide additional details regarding participant consent. In the Methods section, please ensure that you have specified (1) whether consent was informed and (2) what type you obtained (for instance, written or verbal). If your study included minors, state whether you obtained consent from parents or guardians. If the need for consent was waived by the ethics committee, please include this information.*

**Our response:**

We ensure that contest was informed. The participants provided their written informed consent to participate in this study. Additional details were added to the Methods section. Thank you again for your reminder.

**See pp. 461-464**

Ethical review and approval were not required for the study on human participants in accordance with the local legislation and institutional requirements. The participants provided their written informed consent to participate in this study.

3. Thank you for stating the following financial disclosure:

“This research was supported by Hainan Province Natural Science Foundation of China (Grant No. 722QN291). Xiao H.W. received this award.”

Please state what role the funders took in the study.  If the funders had no role, please state: "The funders had no role in study design, data collection and analysis, decision to publish, or preparation of the manuscript."

If this statement is not correct you must amend it as needed.

Please include this amended Role of Funder statement in your cover letter; we will change the online submission form on your behalf.

**Our response:**

We have added the statement in the cover letter.

4. We note that you have indicated that data from this study are available upon request. PLOS only allows data to be available upon request if there are legal or ethical restrictions on sharing data publicly. For more information on unacceptable data access restrictions, please see http://journals.plos.org/plosone/s/data-availability#loc-unacceptable-data-access-restrictions.

In your revised cover letter, please address the following prompts:

a) If there are ethical or legal restrictions on sharing a de-identified data set, please explain them in detail (e.g., data contain potentially sensitive information, data are owned by a third-party organization, etc.) and who has imposed them (e.g., an ethics committee). Please also provide contact information for a data access committee, ethics committee, or other institutional body to which data requests may be sent.

b) If there are no restrictions, please upload the minimal anonymized data set necessary to replicate your study findings as either Supporting Information files or to a stable, public repository and provide us with the relevant URLs, DOIs, or accession numbers. For a list of acceptable repositories, please see http://journals.plos.org/plosone/s/data-availability#loc-recommended-repositories.

We will update your Data Availability statement on your behalf to reflect the information you provide.

**Our response:**

We have provided the data in the supporting information.

5. PLOS requires an ORCID iD for the corresponding author in Editorial Manager on papers submitted after December 6th, 2016. Please ensure that you have an ORCID iD and that it is validated in Editorial Manager. To do this, go to ‘Update my Information’ (in the upper left-hand corner of the main menu), and click on the Fetch/Validate link next to the ORCID field. This will take you to the ORCID site and allow you to create a new iD or authenticate a pre-existing iD in Editorial Manager. Please see the following video for instructions on linking an ORCID iD to your Editorial Manager account: <https://www.youtube.com/watch?v=_xcclfuvtxQ>

**Our response:**

Thank you. We did make sure that corresponding author link his ORCID to the editorial manager account.

6. Your ethics statement should only appear in the Methods section of your manuscript. If your ethics statement is written in any section besides the Methods, please move it to the Methods section and delete it from any other section. Please ensure that your ethics statement is included in your manuscript, as the ethics statement entered into the online submission form will not be published alongside your manuscript.

**Our response:**

Thank you for this comment. We have added the ethical concern in the Method section.

See pp. 223-226

**Ethical Concern**

Ethical review and approval were not required for the study on human participants in accordance with the local legislation and institutional requirements. The participants provided their written informed consent to participate in this study.

7. Please include captions for your Supporting Information files at the end of your manuscript, and update any in-text citations to match accordingly. Please see our Supporting Information guidelines for more information: <http://journals.plos.org/plosone/s/supporting-information>.

**Our response:**

Thank you for this comment. We have listed the supporting information captions and uploaded the materials.

8. Please review your reference list to ensure that it is complete and correct. If you have cited papers that have been retracted, please include the rationale for doing so in the manuscript text, or remove these references and replace them with relevant current references. Any changes to the reference list should be mentioned in the rebuttal letter that accompanies your revised manuscript. If you need to cite a retracted article, indicate the article’s retracted status in the References list and also include a citation and full reference for the retraction notice.

**Our response:**

We have checked the references and removed the retracted articles.

**Responses to Reviewer # 1 Comments**

1. *Good work. I've read it with interest. The paper is well written and nicely thought out. With a minor revision, I believe this paper can be accepted. My only reservation is that a lack of reference. Related research is not well outlined nor considered critically. Thus, I urge the authors to redo literature review. I suggest the following works for your new references:*

*Hameleers, M., Park, Y., Diakopoulos, N., Helberger, N., Lewis, S., Westlund, O. & Baumann, S. (2022). Countering algorithmic bias and disinformation and effectively harnessing the power of AI in media. Journalism & Mass Communication Quarterly, 99(4), 887-907. https://doi.org/10.1177/10776990221129245
Shin, D. (2023). Embodying algorithms, enactive AI, and the extended cognition: You can see as much as you know about algorithm. Journal of Information Science, 49(1), 18-31. http://doi:10.1177/0165551520985495
Shin, D. (2022). The perception of humanness in conversational journalism: An algorithmic information-processing perspective. New Media & Society, 24(12), 2680-2704. http://doi:10.1177/1461444821993801
Lim, J., Ahmad, N., & Ibarahim, M. (2022). Understanding user sensemaking in fairness and transparency in algorithms: Algorithmic sensemaking in over-the-top platform. AI & Society.* [*https://doi.org/10.1007/s00146-022-01525-9*](https://doi.org/10.1007/s00146-022-01525-9)

**Our response:**

We sincerely appreciate the valuable comments. We have checked the literature carefully and added the references into the Limitations and Future Directions section in the revised manuscript.

**(See p.17)**

Finally, our research is limited to traditional conditions. However, technological innovation has brought changes to our life, especially the usage of AI. Past researches indicated that algorithmic consumption was associated with users’ cognition and perception [49-52]. Future research could further explore the influence of AI on individuals’ general categorization tendency.

**Responses to Reviewer # 2 Comments**

1. *Abstract is well structured and identifies clearly major contributions, although the method applied is missing. Very good read, well structured, and with strong robustness of results that support the study.*

**Our response:**

Thank you for your excellent comments! Your points are well taken. We have included the methods used in the research in the abstract, where changes could be found in the revised manuscript.

**(See p.1)**

**Abstract**

Prior studies revealed varying effects of resource scarcity on individuals’ general categorization tendency. However, little is known about when and why such differences occur. Based on the self-regulatory model of resource scarcity, we examine whether resource scarcity generates higher or lower general categorization tendency depending on the perceived mutability of the resource discrepancy. We conducted two online experiments to test the hypotheses. The results affirmed that when individuals consider the resource discrepancy to be mutable, they are more likely to seek abundance to compensate for resource scarcity, thus reducing their general categorization tendency. In contrast, perceiving the scarcity as immutable triggers the intention to restore a sense of control undermined by the scarcity, increasing individuals’ general categorization tendency. Our findings provide insights into the downstream consequences of resource scarcity and offer significant managerial implications for coping strategies.

2. *Overall, there are small format issues that need to be corrected.*

*(1) Figure 2 is not mentioned in the document.*

*(2) 5 should be Conclusions and 5.1 renamed as Discussion and merged with 4.3.*

*(3) In 5.1 the authors could elaborate more on the results' discussion and clearly relate to the hypothesis formulated in 2.2.*

(4) *In 5.3 Limitations and Future Directions explain better the difficulties along the study and how the authors have surpassed them and how they plan to tackle them in the future.*

**Our response:**

Thank you for raising these issues. We recognized that there were indeed formatting and writing issues in the original manuscript. Thus, we corrected the formatting issues.

(1) In the revised manuscript, we have added the missing content.

**(See p.13)**

A 2 (resource scarcity vs. abundance) × 2 (mutability: low vs. high) ANOVA on categorization tendency revealed a significant interaction between scarcity and mutability (*F* (1, 196) = 13.52, *p* < .001, *η*^2^ = 0.06). Figure 2 shows the results. Specifically, in the low mutability condition…

|  | scarcity | control |
| --- | --- | --- |
| low perceived mutability | 5.145 | 4.655 |
| high perceived mutability | 3.71 | 4.495 |

**Fig 2. Interaction effect of resource scarcity and perceived mutability on general categorization tendency**

(2) We have renamed 5 into **General Discussion**. Meanwhile, 5.1 discussion was renamed as conclusion. This section elaborates more on the main conclusion and results' discussion.

**(See pp.14-15)**

(3) We appreciated the valuable comments. According to the suggestions, we have revised this part and elaborated more on the results' discussion.

**(See p.15)**

A growing stream of research clearly shows that resource constraints lead to diverse consequences. However, what is less known is the precise nature of these consequences, including when and why they occur. Our research investigates when and why resource constraints sometimes increase individual’s general categorization tendency (Krosch and Amodio, 2014; Van Kerckhove et al., 2020; Yoon and Kim, 2018) and sometimes decrease the same (Briers and Laporte, 2013; Fan et al., 2019; Park et al., 2020). Drawing on two empirical studies, this research demonstrates that the impact of resource scarcity on general categorization tendencies is contingent upon the mutability of resource scarcity. Specifically, we find that individuals are more likely to enhance their general categorization propensity when they perceive resource scarcity as being low in mutability. This effect is driven by their need to re-establish their sense of personal control, which is threatened by the scarcity. In contrast, when resource scarcity is perceived to be high in mutability, individuals' desire for abundance is exacerbated by the scarcity, making categorization particularly unpleasant. As a result, resource scarcity is expected to lower the tendency for categorization in general. These findings contribute to a deeper understanding of how individuals respond to resource scarcity and its implications for their categorization behavior.

(4) According to the comments, we have further explained the difficulties in the study and supplied the plan to tackle the problem in the future.

**(See p.17)**

This study has several limitations that should be addressed in future research. First, considering the internal validity of the results, our study only applied well-developed manipulations. Future studies should examine the comparative prominence and importance of various types of resource scarcity and investigate how they affect people’s behavior. Second, the approach is limited by the fact that we assessed individuals’ general categorization tendency primarily through self-reports. To further our understanding and increase the external validity of our study, future research should conduct field experiments to test our propositions. Finally, our research is limited to traditional conditions. However, technological innovation has brought changes to our life, especially the usage of AI. Past research indicated that algorithmic consumption was associated with users’ cognition and perception [49-52]. Future research could further explore the influence of AI on individuals’ general categorization tendency.

**Responses to Reviewer # 3 Comments**

1. *Remove “desire for control” and “desire for abundance” from the keywords and replace with ‘self-regulatory model’ and ‘resource discrepancy’ – these would better reflect the content of the paper.*

**Our response:**

We think it is an excellent suggestion. Thank you for raising this point. Following your suggestion, we have changed the corresponding keywords, which definitely better reflect the content of the paper.

**(See p.1)**

**Keywords:** resource scarcity, categorization tendency, perceived mutability, self-regulatory model, resource discrepancy

2. *The opening point in the introduction “Individuals usually perceive resources as scarce” – surely this depends on what the resource is? This is a rather ambiguous statement.*

**Our response:**

Thanks for your valuable comments. Considering the possible ambiguity of this statement, we change the sentence to

**(See p.1)**

Individuals usually perceive resources, including money (Sharma and Alter, 2012), time (Kapoor and Tripathi, 2020), and product (Zhu and Ratner, 2015) as scarce.

3. *Also in the introduction on p2 refers to “general categorization tendency” – this needs defining as a concept.*

**Our response:**

Based on your suggestions, the required definition and explanations have been added to explain the vague concept.

**(See p.1)**

Recent studies examined how the general sense of resource scarcity influences individuals’ general categorization tendency, which refers to a human’s basic motive to seek structures and order by the process of dividing the world into manageable groups of entities (Park et al., 2020; Van Kerckhove et al., 2020; Yoon and Kim, 2018).

4. *In section 2.2 “a psychological sense of decrease” – this does not really mean anything – I suggest this is rephrased to improve the clarity of expression.*

**Our response:**

Based on your suggestion, we have replaced the phrase with “a feeling of reduction”.

**(See p.5)**

This is because categorization evokes a feeling of reduction (i.e., many items are divided into smaller subgroups by categorization).

5. *In section 2.3 – I am not convinced about the religious reference “those who feel less in control tend to believe more strongly that God is in charge” – can this be explained more from a scientific perspective instead?*

**Our response:**

We have rephrased the paragraph and explained more from a scientific perspective.

**(See p.6)**

A lack of personal control engenders an aversive feeling that drives individuals to regain their perception of control to baseline levels (Chen *et al.,* 2017). Previous research demonstrated that people who lack personal control are likely to affirm structured interpretations of the world (Landau *et al.,* 2015; Whitson and Galinsky, 2008). For instance, research showed that low personal control led to increased subsequent beliefs in the existence of a controlling God (Laurin *et al.,* 2008).

6. *Also in 2.3, “individuals who lack control tend to seek structure (Whitson and Galinsky, 2008), which raises preferences for product logos with bounded (vs. unbounded) and numerical forms of point (vs. range) values” – this statement requires more explanation and definition.*

**Our response:**

We have rephrased the paragraph and explained more from a scientific perspective.

**(See p.6)**

In marketing domain, consumers who lack control tend to seek structure through consumption. Lembregts and Pandelaere (2019) show that low personal control leads consumers to prefer and rely more on numerical attributes as a point value, relative to a range format. This occurs because product attributes specified in a point value format strengthen consumers’ belief that the environment is predictable.

7. *In 2.4, “caloric foods” – this should be ‘calorific’ or ‘**high calorie’.*

**Our response:**

We sincerely thank your careful reading. We feel sorry for our mistakes. As suggested by the reviewer, we have corrected the “caloric foods” into “calorific ”

**(See p.7)**

Similarly, Kapoor and Tripathi (2020) suggested that people who experience downward timekeeping are more likely to perceive themselves as resource-scarce, leading to a higher preference for high calorie foods.

8. *At the end of 5.2 “our findings provide an innovative theoretical understanding of coping- mechanisms that might be employed in response to a generalized feeling of resource constraints” – the authors have not really explored coping mechanisms in the discussion sections per se.*

**Our response:**

After serious consideration of your valuable comments and careful examination of our paper, we have recognized that there is really no coping mechanism explored in this section. As a result, we have removed this sentence. Changes can be founded in the revised manuscript.

9. *Finally, in 5.3 – the paper lacks mention of any practical contributions, for instance what does the study conducted show beyond the theory? Are there any managerial implications to consider here? This links back to the lack of practical context of the paper as a whole.*

**Our response:**

Thank you for this comment! We have added the practical implications section.

**(See p.16)**

**Practical implications**

Our findings provide several managerial implications. Our results suggest that marketers could use flexible strategies for their products or services. In certain situations, we should increase or decrease consumers’ categorization perceptions. For example, product displays (e.g., product grouping labels) can bolster consumers’ general categorization tendency. According to our findings, when consumers perceive resource scarcity as being low (high) in mutability, marketers should enhance (reduce) the categorization perceptions of the products or services. In addition, marketers should use the scarcity appeals in proper ways. The effect of scarcity on decision-making is different, since the concept of scarcity is nuanced. We identify the moderating role of mutability. As a result, whether we should activate the perception of resource scarcity depends on the mutability of resource scarcity.

10. *The final statements regarding conflict of interest etc. appear in numbered sections – 6-9 – I do not believe these should require a numbered section.*

**Our response:**

Thanks for your comments. Based on your suggestion, we have removed these numbers to optimize the structure of the paper. Traces of alternations can be found in the resubmitted manuscript clearly.

Based on your valuable comments, we have tried our best to improve the manuscript and made some changes marked in red in revised paper which will not influence the content and framework of the paper. We appreciate for your serious work, and hope correction will meet with approval. Once again, thanks for your suggestions.

Yours truly,

The authors
