# Supplementary material for: Impact of resource scarcity on general categorization tendency: The moderating role of perceived mutability
Source: PLoS One. 2023 Aug 23;18(8):e0286619. doi: 10.1371/journal.pone.0286619 (PMC10446183; doi:10.1371/journal.pone.0286619)
Supplement: Attachment — Submitted filename: renamed_a1fc5.docx [file pone.0286619.s004.docx]

**Author’s Response to Reviewers**

Dear Editor:

Re: “Impact of Resource Scarcity on General Categorization Tendency: The Moderating Role of Perceived Mutability” (Manuscript: PONE-D-23-02056)

On behalf of all the contributing authors, I would like to express our sincere appreciation for your letter and the reviewers’ comments on our paper.

Each comment from all reviewers has been carefully considered and responded to. To make it easier for you to read, we have organized our feedback in the following format. The reviewers' comments are listed in *italicized font*, and specific concerns have been specifically numbered. Our replies are given in normal font, in addition, corrections and additions to the manuscript are given in red font.

Thank you again for taking the time to carefully review our manuscript. We are grateful to have you as the editor of our paper.

Below we present our responses to the specific issues raised by each reviewer.

Yours truly,

The authors

Response to the review 3:

1. *Since I had not previously had sight of figures 1 and 2 until the revised manuscript had been submitted I have just one minor point – figure 2 is missing a label on the y-axis – what is the 1.00 to 7.00 scale measuring?*

**Our response:**

Thanks for your careful review and reminder, we have rechecked and added the label on the y-axis of Figure 2 to exactly express the meaning of the 1.00 to 7.00 scale measuring. Changes could be found clearly in our resubmitted manuscript.

(See p.13 and line.309)

general categorization tendency

|  | scarcity | control |
| --- | --- | --- |
| low perceived mutability | 5.145 | 4.655 |
| high perceived mutability | 3.71 | 4.495 |

**Fig 2.** **Interaction effect of resource scarcity and perceived mutability on general categorization tendency.**
